# The Critical Balance: Understanding Blood Pressure and Intracranial Hemorrhage Association in Endovascular Stroke Therapy

**DOI:** 10.1007/s00270-023-03655-z

**Published:** 2024-03-07

**Authors:** Christoph J. Maurer, Ansgar Berlis

**Affiliations:** Diagnostic and Interventional Neuroradiology, University Hospital Augsburg, Augsburg, Germany

Managing blood pressure before, during, and after treating acute ischemic stroke presents a significant challenge. On one hand, elevated blood pressure helps maintain collaterals in the context of an acute vessel occlusion. On the other hand, high blood pressure, particularly during intravenous thrombolysis, carries the risk of intracranial hemorrhage, and low blood pressure can accelerate the breakdown of the collateral blood supply, leading to larger infarcts [1]. Post-therapy, both high blood pressure and high variability in blood pressure lead to poorer outcomes and increased risks of intracranial hemorrhage, especially when anticoagulant or antiplatelet drugs are necessary, such as in cases of extracranial stenting [2]. The nuances of blood pressure management during endovascular therapy are even less understood. Theoretically, maintaining elevated blood pressure is desirable as long as the vessel remains occluded, while aiming for lower or normal blood pressure is advisable once the vessel is recanalized, due to the increased risk of reperfusion injury. However, even highly individualized approaches to blood pressure management during endovascular therapy have shown no significant benefit over standardized regimens targeting systolic blood pressure [3].

Moreover, assessing the risk of hemorrhage after endovascular therapy for acute ischemic stroke typically involves standard CT scans 24 h post-therapy, where blood–brain barrier disruption can lead to hemorrhage overestimation due to overlapping aspects with contrast enhancement.

Robbe et al. address these topics in their study in the current issue [4]. Firstly, they examined patients within 3 h after endovascular therapy using dual-energy CT to differentiate between contrast enhancement and hemorrhage. Secondly, they correlated the findings with blood pressure measurements taken during endovascular therapy. They discovered an association between elevated blood pressure and parenchymal hemorrhage. Interestingly, extraparenchymal hemorrhage, not uncommon after endovascular therapy, was not associated with higher blood pressure but rather with technical aspects of the procedure.

However, as is often the case, correlation does not imply causation, and these results should be interpreted cautiously. While patients with extraparenchymal hemorrhage did not exhibit higher rates of elevated blood pressure, supporting the hypothesis that hemorrhage alone is not a cause for excess blood pressure, patients with parenchymal hemorrhage had a higher rate of preexisting hypertension (69% vs. 50%) and previous antiplatelet medication use (62% vs. 33%) compared to the non-hemorrhage group. These patients might inherently possess a higher risk of microvascular injury due to their preexisting conditions and blood pressure adjustment may be more challenging due to potential chronic changes in cerebral autoregulation.

What, then, should the interventionalist and their anesthesiological team do in the light of this complexity? First, invasive blood pressure monitoring, readily obtainable via the femoral sheath, is highly advisable for responding to significant blood pressure changes. Second, a pragmatic approach targeting systolic blood pressure between 140 and 180 mmHg appears safe and feasible, yielding outcomes similar to those from highly individualized approaches [3]. Third, dual-energy CT, and more recently, spectral or photon counting CT, provide more accurate and distinct information on post-endovascular therapy outcomes, especially in differentiating between contrast extravasation due to blood–brain barrier disruption and actual hemorrhage. This precision offers greater certainty on how to respond to blood pressure changes in the days following thrombectomy.

The extent to which the findings of the current study will be impactful remains uncertain, but this data may be valuable in stratifying and individualizing blood pressure management in the future and in designing subsequent trials on this topic.
